# Analysis of control strategies for VIVA OpenHBM with active reflexive neck muscles

**DOI:** 10.1007/s10237-022-01616-y

**Published:** 2022-08-04

**Authors:** I Putu A. Putra, Robert Thomson

**Affiliations:** Division of Vehicle Safety, Department of Mechanics and Maritime Sciences, Chalmers University of Technology (Campus Lindholmen), Hörselgången 4, 41296 Gothenburg, Sweden

**Keywords:** Finite element, Human body model, Muscle controller, Muscle spindle, Vestibular system, Whiplash injury

## Abstract

Modeling muscle activity in the neck muscles of a finite element (FE) human body model can be based on two biological reflex systems. One approach is to approximate the Vestibulocollic reflex (VCR) function, which maintains the head orientation relative to a fixed reference in space. The second system tries to maintain the head posture relative to the torso, similar to the Cervicocolic reflex (CCR). Strategies to combine these two neck muscle controller approaches in a single head-neck FE model were tested, optimized, and compared to rear-impact volunteer data. The first approach, Combined-Control, assumed that both controllers simultaneously controlled all neck muscle activations. In the second approach, Distributed-Control, one controller was used to regulate activation of the superficial muscles while a different controller acted on deep neck muscles. The results showed that any muscle controller that combined the two approaches was less effective than only using one of VCR- or CCR-based systems on its own. A passive model had the best objective rating for cervical spine kinematics, but the addition of a single active controller provided the best response for both head and cervical spine kinematics. The present study demonstrates the difficulty in completely capturing representative head and cervical spine responses to rear-impact loading and identified a controller capturing the VCR reflex as the best candidate to investigate whiplash injury mechanisms through FE modeling.

**Supplementary Information:**

The online version contains supplementary material available at 10.1007/s10237-022-01616-y.

## Introduction

Closed loop-based control has been used to control muscle activations in finite element (FE) human body models (HBMs) and has been shown to increase their biofidelity (Östh et al. 2015). In the closed-loop approach, muscle activation is based on a sensor and control system that approximates one or several human feedback systems. An active HBM (AHBM) with a closed-loop approach can generally be used in a broader range of applications than those with an open-loop control method as the latter is defined a-priori for specific load cases. AHBMs with closed-loop muscle control have been successfully used to study occupant kinematics in different loading conditions (Östh et al. 2012a, 2014b, 2014a; Iwamoto and Nakahira 2015; Kleinbach 2019; Devane et al. 2019; Martynenko et al. 2019; Putra et al. 2019, 2020; Correia et al. 2021).

AHBMs are being developed to provide a better understanding of head-neck kinematics during collisions causing whiplash. The head-neck kinematics of the occupant during rear-impacts are found to be influenced by cervical muscle activity (Brault et al. 2000; Siegmund et al. 2003; Blouin et al. 2006; Dehner et al. 2013; Mang et al. 2015). In terms of volume, cervical muscles are a significant part of the neck structure. It is also postulated that neck muscles could influence the whiplash injury risk by directly or indirectly affecting other anatomical structures of the neck (Siegmund et al. 2009). The high societal cost and prevalence of whiplash injuries (Bannister et al. 2009) motivates further research of its injury mechanisms.

Several FE models have been developed with active neck muscle controllers to simulate neck muscle reflexes. Several models called SAFER A-HBM (Östh et al. 2012a, 2014b, 2014a; Ólafsdóttir et al. 2019b), THUMS version 5 (Iwamoto and Nakahira 2015), VIVA OpenHBM (Kleinbach 2019; Putra et al. 2019, 2020)**,** GHBMC (Correia et al. 2021), THUMS TUC-VW AHBM and A-THUMS-D (Martynenko et al 2021) have been used to model human reflex mechanisms for controlling the cervical spine muscle activation. However, all models used either a single controller, assumed simple addition (Ólafsdóttir et al. 2019b) or a limited combination using an IF function (Correia et al. 2021) of different controllers to control the neck muscle activations.

Previous work using VIVA OpenHBM (Putra et al. 2019) implemented two closed-loop approaches to control the neck muscles. The first approach was the Angular-positioned Feedback (APF) controller. The APF controller activates the neck muscles to maintain the head orientation relative to space. It was intended to approximate the Vestibulocollic reflex (VCR) in humans. The second approach was called a Muscle-length Feedback (MLF) controller. It was developed to have a similar function as the Cervicocolic reflex (CCR), which keeps the head posture relative to the torso. After the controllers were tuned, both active models were then compared to published volunteer data. Although the agreement of simulated head kinematics with volunteer data was greater in a model with an APF controller, the authors recommended that both active muscle controllers should be combined to develop a more biofidelic model to limit non-representative cervical spine kinematics. Similar research has established that the muscle controller approximating the stretch reflex from muscle spindle could be beneficial to reduce the cervical spine buckling caused by the controller representing the vestibular system (Ólafsdóttir et al. 2019a). However, it is unclear whether combining two muscle controllers could reduce the non-representative cervical spine kinematics (buckling) occurred in the previous works of the VIVA OpenHBM Model.

The original model used in (Putra et al. 2019, 2020) was previously validated against cadaver (PHMS) responses. The model was then tuned with an active muscle controller to match the volunteer responses during impact. However, in those studies, a fixed muscle co-contraction activation level was assumed. Therefore, it wasn’t clear whether the model on those studies could stay upright under gravitational acceleration. Maintaining an upright head position with the accompanying muscle and vertebral loads from posture muscle and gravitational loading are essential to represent a volunteer’s initial equilibrium position before impact.

The main goal of the present study was to understand the best control strategy for simulating low-speed rear impacts. The two muscle control strategies (APF and MLF) can be implemented individually or in implementations where both systems act simultaneously in a single head-neck finite element (FE) model. In addition, optimization of the muscle co-contraction was also proposed and applied to the model to develop a model that can stay upright under gravitational acceleration before horizontal loads are applied. The effects of different approaches to combine active muscle controllers, including with and without muscle co-contraction, should be compared to find the best reproduction of kinematic data from volunteer testing.

## Materials and method

### The 50th percentile female head-neck VIVA OpenHBM with simplified cervical spine

VIVA OpenHBM was developed to represent the 50th percentile female population and was specifically developed to study whiplash injuries (Östh et al. 2017a, 2017b). The passive neck muscle modeling of VIVA OpenHBM was described in Östh et al. (2017b). The neck muscles of the VIVA OpenHBM model were implemented based on the Hill muscle model (LS-DYNA *MAT_156/*MAT_MUSCLE) with physiological cross-sectional area (PCSA) from Borst et al. (2011)**.** The origin and insertions of the muscles were based on anatomical descriptions from Gray and Standring (2008). There are 129 1D Truss muscle elements to represent 34 muscles (Östh et al. 2017b). Two types of VIVA OpenHBM models are available: a model with a detailed cervical spine and a simplified cervical spine model. The simplification in the cervical spine was done by removing non-muscular soft tissues and replacing them with compliant joints (axial rotational, lateral bending, and flexion–extension joints) based on in-vitro human subject response data (Östh et al. 2017a). In total, there were 21 curves describing the compliant joint properties from C1 (the first cervical spine vertebra) to C7 (the seventh cervical spine vertebra). This simplification saves 39% of computational time with no significant change in the CORA rating score for the rear impact cases (Östh et al. 2017a). In the present study, the VIVA OpenHBM head-neck model with simplified cervical spine and adjusted cervical spine curvature (Putra et al. 2020) was used.

### Developing a head-neck model to stay upright under gravitational acceleration

A model that can stay upright under gravitational acceleration was achieved by adjusting and optimizing the muscle co-contraction level. The muscle co-contraction is defined as the simultaneous activation of agonistic and antagonistic muscles and has been known to contribute to maintaining spinal stability (Lee et al. 2006). The neck muscle co-contraction should maintain its stability and directly keep the head in an upright position under gravitational acceleration. In the present study, the neck muscle of the VIVA OpenHBM model was divided into eight groups of muscles based on Ólafsdóttir et al. (2019b) before optimizing the co-contraction level (Table 1). The optimization simulations were conducted using the LS-OPT software with the optimization objective of zero pitch rotation of the Head Centre of Gravity (CG). For the optimization method, the Metamodel-based Optimization using Sequential Response Surface Method (SRSM) with Domain Reduction was selected as the optimization method in LS-OPT. In addition, the Linear Polynomial Metamodel with D-optimal point selection was adopted as the metamodel. The algorithm was based on The Hybrid SA (Simulated Annealing + Leapfrog Optimizer for Constrained Minimization). This optimization strategy produces 14 simulation points per iteration. The total number of iterations was set to 10. See Stander et al. (2015) for the details of Optimization strategies used in this study.

**Table 1:** Setup for muscle co-contraction optimization

Muscle name	Muscle group	Optimization range	Simulation time
Sternocleidomastoid (SCM), Scalenus Posterior, Scalenus Medius, Scalenus Anterior, Rectus Capitis Anterior	SCM group	0.01–0.05 (1–5%)	500 ms
Sternohyoid (STH), Sternothyroid, Omohyoid, Longus Colli Superior Oblique, Longus Colli Vertical, Longus Colli Inferior Oblique, Longus Capitis	STH group	0.01–0.05 (1–5%)	500 ms
Levator Scapulae (LS)	LS group	0.01–0.05 (1–5%)	500 ms
Trapezius (Trap)	Trap group	0.01–0.05 (1–5%)	500 ms
Semispinalis Capitis (SCap), Rectus Capitis Posterior Minor, Recturs Capitis Posterior Major, Obliqus Capitis Superior	SCap group	0.01–0.05 (1–5%)	500 ms
Semispinalis Cervicis (SCerv), Semispinalis Thoracis, Splenius Capitis, Splenius Cervicis, Erector Spinae Longissimus Capitis, Erector Spinae Longissimus Cervics, Erector Spinar Iliocostalis Cervicis	SCerv group	0.01–0.05 (1–5%)	500 ms
Multifidus Cervicis (CM-C4)	CM-C4 group	0.01–0.05 (1–5%)	500 ms
Multifidus Cervicis, Obliqus Capitis Inferior, Rectus Capitis Lateralis	CM-C6 group	0.01–0.05 (1–5%)	500 ms

### Volunteer data

The published volunteer data from Sato et al. (2014) were utilized to optimize to derive the active muscle controller gains and evaluate the model's performance. The data of Sato et al. (2014) consisted of head center of gravity (C.G) linear displacements (x- and z-direction), head C.G rotational displacement (in the y-direction), and cervical spine (C1-C7) rotational y-displacements. The data were derived based on a low-speed rear impacts with two female volunteers. The volunteers were seated in a rigid seat without a headrest and were impacted from behind. The impact acceleration pulse produced a delta velocity of 5.8 km/h with a peak acceleration of 42 m/s^2^. All loads were applied in the sagittal plane.

### Active muscle controller combination and optimization

**Fig. 1:**
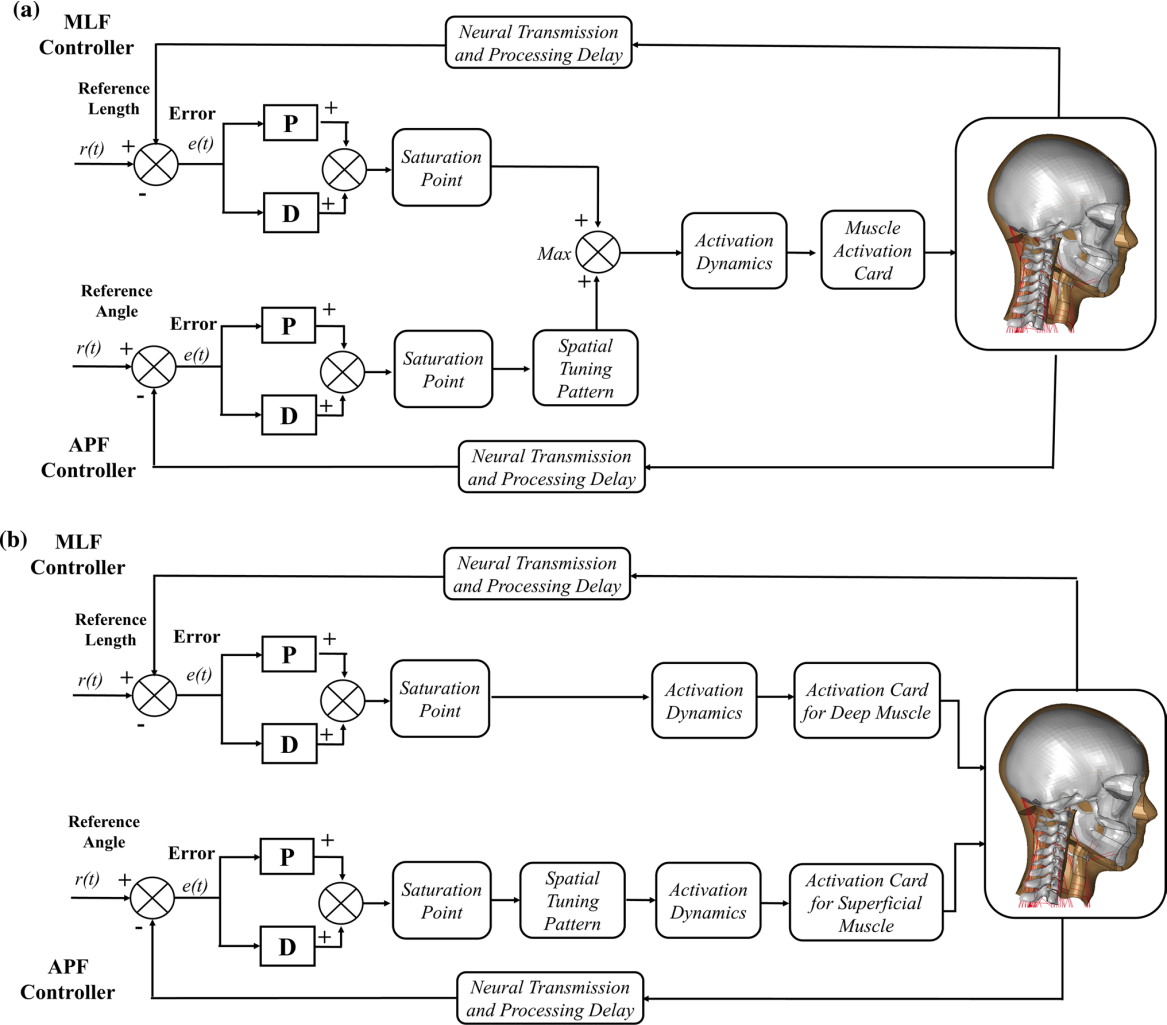
**a** Combined-control approach and **b** Distributed-control approach

Two approaches were studied to combine the APF and MLF controller (Fig. 1). The first approach assumed that both APF and MLF controllers were controlling the activation of all neck muscles (based on the review from Armstrong et al., 2008). However, since in LS-DYNA *MAT_156/*MAT_MUSCLE, only one activation card is available, the stronger output signal between APF and MLF controller was selected using the Min–Max function at each moment of time. Consequently, for the impact duration of 300 ms, the activation signal for each muscle element was based on the combination of APF and MLF signals. This first approach is called a Combined-Control approach (Fig. 1a). In the second approach, the APF controller was used to regulate the activation of the superficial muscles. Meanwhile, the deep neck muscles were controlled by the MLF controller. The second approach was motivated by the higher densities of muscle spindles found in the deep cervical muscles (Amonoo-Kuofi 1983; Liu et al. 2003). The grouping of deep and superficial neck muscles was based on Borst et al. (2011). The second approach is denoted the Distributed-Control approach (Fig. 1b).

The optimum gain of each proposed method was derived by conducting optimization-based parameter identification using LS-OPT (Table 2), similar to that used for the co-contractions. These optimizations were aimed to identify the optimum parameters for the APF and MLF controllers with the objective to match the volunteer head linear (x- and z-) and rotational y-displacement (or pitch) as well as cervical spine (C1–C7) rotational y-displacements (details in Online Resource 1). Those parameters were the Proportional gain (KP), Derivative gain (KD), and the neural transmission and processing time delay (TND). Meanwhile, other parameters were kept constant following Putra et al. (2020). For the APF controller, the Proportional gain (KP), Derivative gain (KD), and the neural transmission and processing time delay (TND) will be referred as the KPA (Proportional gain APF), KDA (Derivative gain APF) and TNDA (the neural transmission and processing time delay APF). Whereas for the MLF controller, the Proportional gain (KP), Derivative gain (KD), and the neural transmission and processing time delay (TND) will be referred as the KPM (Proportional gain MLF), KDM (Derivative gain MLF) and TNDM (the neural transmission and processing time delay MLF). In the current study, the initial value of KPA, KDA, and TNDA was based on Putra et al. (2020). But for the MLF controller, initialization values for optimization were based on Putra et al. (2019). The upper and lower range of KP (KPA and KPM) and KD (KDA and KDM) for both controllers were set as 0.01 to 100, based on the author's experience. Previously in Putra et al. (2019 and 2020), optimization ranges from 0.601–40 were used for the KP, and 5–412.62 were set for the KD. However, since the present work introduced the different co-contraction levels and combinations of two separate controllers, different ranges were tested as pre-studies. As a result, it was found that a range of 0.01 to 100 could cover the optimization search space. The range of the TNDA was set from 3.5 ms (Rosengren and Colebatch 2018) to 20 ms (Ólafsdóttir et al. 2019b). The ranges for the TNDM were from 10 ms (Ólafsdóttir et al. 2019b) to 54 ms (Putra et al. 2019). For the active muscle controller combination optimization, 11 simulation points per iteration were produced with the total number of iterations set to 10.

**Table 2:** Optimization range

Controller	Parameter	Symbol	Unit	Initial value	Optimization range
APF controller	Proportional gain	KPA	%contraction/rad	6	0.01–100
	Derivative gain	KDA	%contraction/rad ms-^1^	5	0.01–100
	Neural transmission and processing delay	TNDA	ms	20	3.5–20
MLF controller	Proportional gain	KPM	%contraction/mm	0.5	0.01–100
	Derivative gain	KDM	%contraction/mm ms-^1^	7	0.01–100
	Neural transmission and processing delay	TNDM	ms	53	10–54

Besides conducting optimizations for the Distributed and Combined control approach, optimizations were also undertaken to derive optimum parameters for the APF and MLF controller only implementations. This was intended to compare the performance of the models with two combined controllers and single controller implementations.

### Quantitative ratings evaluation

Correlation Analysis (CORAplus) software 4.0.4 (Gehre et al. 2009) was used to conduct an objective rating evaluation with rating scale as shown by Table 3. This evaluation was aimed to quantify the similarities of the model and volunteer kinematics responses. The head kinematics were compared for the whole duration of the simulation (300 ms). Due to limitations in the X-Ray field of view, the volunteer cervical spine kinematics were only compared from 0 to 200 ms with the last 20 ms approximated by extrapolation (Online Resource 1). Default corridors of CORA (5% inner limit and 50% outer limits) were used.

**Table 3:** Correlation Rating Scale (ISO/TR 9790, 1999)

Rating	Correlation score
Excellent	0.860–1.000
Good	0.650–0.860
Fair	0.440–0.650
Marginal	0.260–0.440
Unacceptable	0.000–0.260

### Software and computational environment

All simulations were run using LS-DYNA R9.2.0. MPP double-precision. LS-PrePost 4.8 (64-bit) and OriginPro 2019(64-bit) were used as pre- and post-processing software.

## Results

### Muscle co-contraction-level optimization

**Fig. 2:**
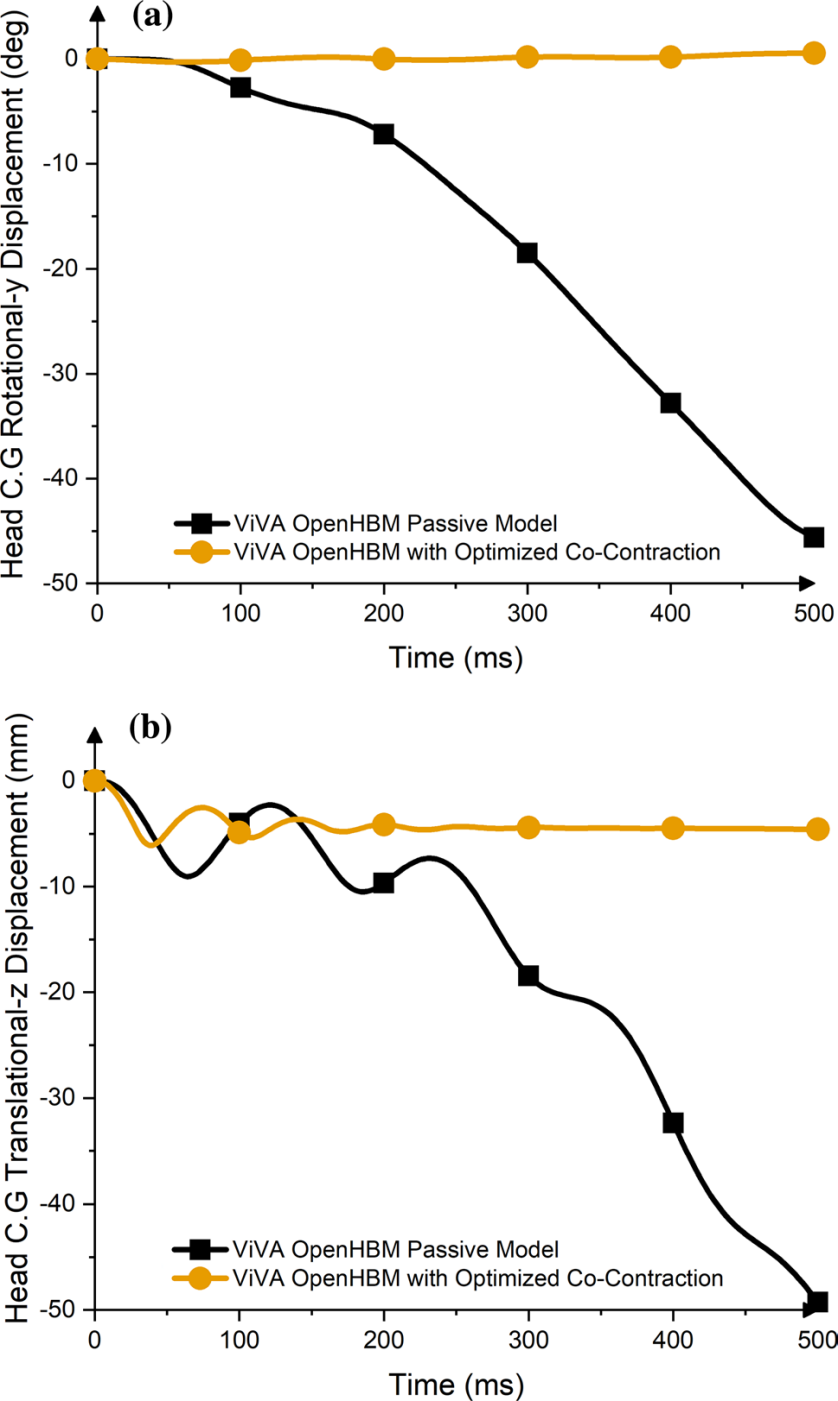
Head displacement comparison between original passive model and model with optimized co-contraction level; **a** Head C.G Rotational y-Displacement. **b** Head C.G Translational z-Displacement

The results of the muscle co-contraction optimization are presented in Table 4. It was found that Multifidus Cervicis, Obliqus Capitis Inferior, Rectus Capitis Lateralis (CM-C6 group) muscles had the highest co-contraction level (4.94%), meanwhile the lowest co-contraction level was assigned to Scap group (Semispinalis Capitis, Rectus Capitis Posterior Minor, Recturs Capitis Posterior Major, Obliqus Capitis Superior muscles). It was also observed that the model with optimized co-contraction-level neck muscle could maintain its posture under the gravity loading after 200 ms based on the head Centre of Gravity (C.G) rotational-y displacement and vertical-z displacement (Fig. 2).

**Table 4:** Muscle co-contraction-level optimization results

Muscle name	Muscle group	Optimized co-contraction level
Sternocleidomastoid (SCM), Scalenus Posterior, Scalenus Medius, Scalenus Anterior, Rectus Capitis Anterior	SCM group	0.0105 (1.05%)
Sternohyoid (STH), Sternothyroid, Omohyoid, Longus Colli Superior Oblique, Longus Colli Vertical, Longus Colli Inferior Oblique, Longus Capitis	STH group	0.0478 (4.78%)
Levator Scapulae (LS)	LS group	0.0131 (1.31%)
Trapezius (Trap)	Trap group	0.0480 (4.80%)
Semispinalis Capitis (SCap), Rectus Capitis Posterior Minor, Recturs Capitis Posterior Major, Obliqus Capitis Superior	SCap group	0.0104 (1.04%)
Semispinalis Cervicis (SCerv), Semispinalis Thoracis, Splenius Capitis, Splenius Cervicis, Erector Spinae Longissimus Capitis, Erector Spinae Longissimus Cervics, Erector Spinar Iliocostalis Cervicis	SCerv group	0.0196 (1.96%)
Multifidus Cervicis (CM-C4)	CM-C4 group	0.0374 (3.74%)
Multifidus Cervicis, Obliqus Capitis Inferior, Rectus Capitis Lateralis	CM-C6 group	0.0494 (4.94%)

### Active muscle controller optimization

The results of KPA, KDA, TNDA, KPM, KDM, TNDM and their different configurations are presented in Table 5. The lowest Proportional gain was specified for the MLF controller (0.26%contraction/mm). Meanwhile, the highest Proportional gain was identified in the APF controller when it’s combined with the MLF Controller using the Combined-Control approach (92.30%contraction/rad). The KP of the APF controller was always higher than the KP from the MLF controller except when the MLF controller only acted on the neck’s deep muscles.

**Table 5:** Optimum parameters of various controller with optimized co-contraction

Optimization name	Controller	Symbol	Unit	Optimum parameter with optimized co-contraction
APF + Co(Angular-positioned Feedback + Co-Contraction)	APF controller	KPA	%contraction/rad	0.97
		KDA	%contraction/rad ms-^1^	32.67
		TNDA	Ms	12.67
MLF + Co (Muscle-length Feedback + Co-Contraction)	MLF controller	KPM	%contraction/mm	0.26
		KDM	%contraction/mm ms-^1^	0.186
		TNDM	ms	17.51
CC + Co (Combined-Control + Co-Contraction)	APF controller	KPA	%contraction/rad	92.30
		KDA	%contraction/rad ms-^1^	41.04
		TNDA	ms	3.50
	MLF controller	KPM	%contraction/mm	59.66
		KDM	%contraction/mm ms-^1^	5.36
		TNDM	ms	17.37
DC + Co (Distributed-Control + Co-Contraction)	APF controller	KPA	%contraction/rad	7.505
		KDA	%contraction/rad ms-^1^	18.34
		TNDA	ms	5.024
	MLF controller	KPM	%contraction/mm	69.534
		KDM	%contraction/mm ms-^1^	16.865
		TNDM	ms	16.48

The lowest Derivative gain value was found for the model with MLF Controller only (0.186%contraction/mm ms-1). The highest Derivative gain was the APF controller in the Combined-Control approach (41.04%contraction/rad ms-1). The slowest (17.51 ms) and the fastest (3.50 ms) neural transmission and processing delay were assigned to APF Controller in the Combined Control approach and the model with MLF controller, respectively.

### Head and cervical spine kinematics comparison

**Fig. 3:**
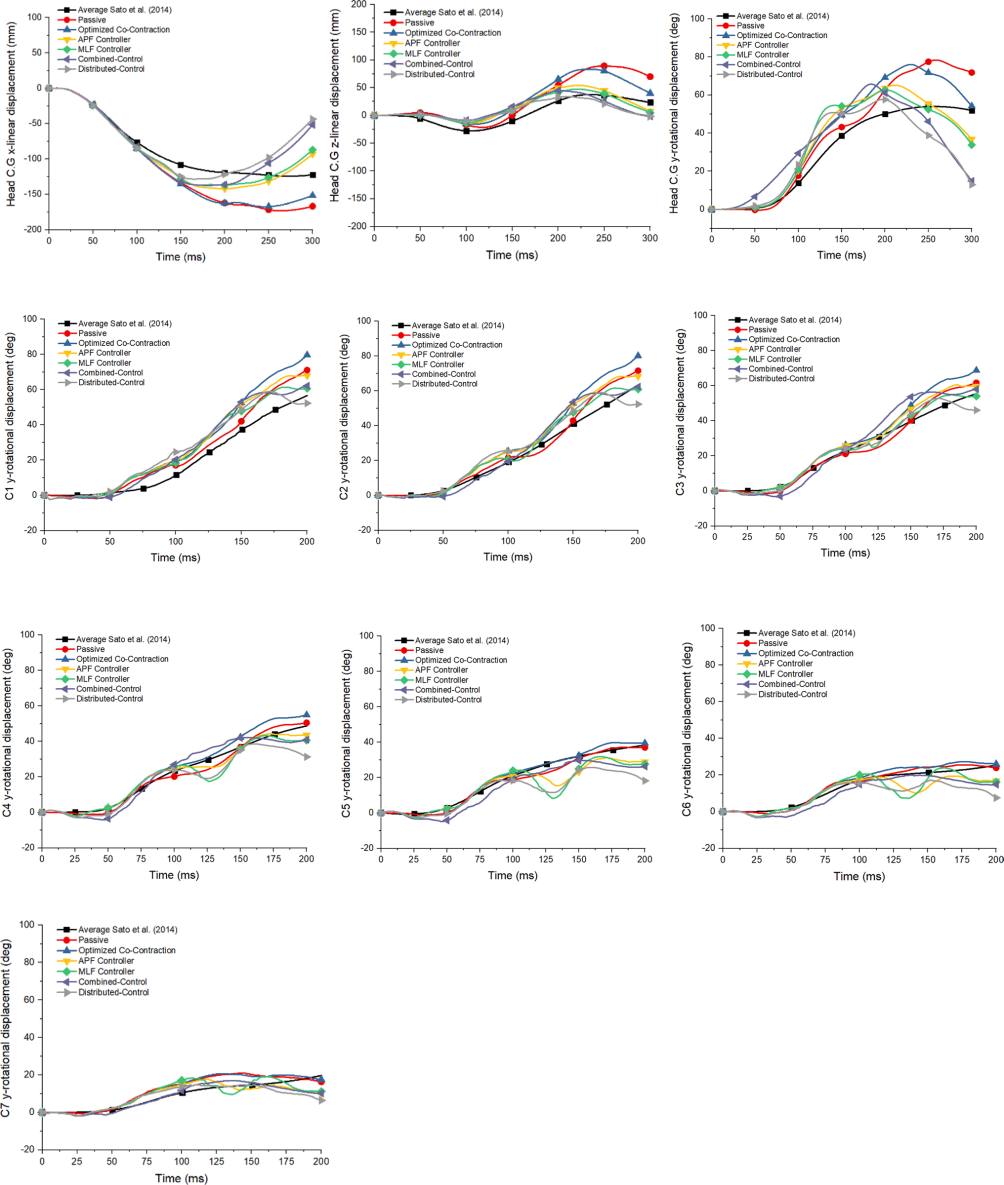
Comparison of head displacements and cervical vertebral rotational y-displacement

Head displacements for the three degrees of freedom were compared for all controller implementations (Fig. 3). For head C.G x-displacements, models with only APF or MLF controllers were those that could best follow the volunteer’s head kinematics. This is reflected in the CORA scores in Table 6. Models with combined and distributed controllers over-predicted the displacements at 200 ms (optimization reference period). Similar trends were also observed in the head vertical z-displacement. When the head C.G rotations were compared, more rebound was observed in the active models than the passive model. The model with Distributed-Control had the worst rebound performance.

**Table 6:** CORA score of head and cervical spine kinematics with various controller

Kinematics	Passive	Co	APF + Co	MLF + Co	CC + Co	DC + Co
HCG-x	0.701	0.711	0.864	0.885	0.822	0.834
HCG-z	0.426	0.396	0.465	0.499	0.406	0.402
HCG-ry	0.749	0.719	0.780	0.772	0.660	0.719
**Average HCG**	0.625	0.609	0.703	0.719*	0.629	0.652
C1-ry	0.844	0.731	0.750	0.803	0.792	0.774
C2-ry	0.926	0.829	0.865	0.929	0.908	0.893
C3-ry	0.936	0.862	0.914	0.969	0.864	0.956
C4-ry	0.966	0.906	0.966	0.904	0.899	0.857
C5-ry	0.957	0.967	0.824	0.777	0.808	0.703
C6-ry	0.932	0.884	0.794	0.766	0.777	0.687
C7 -ry	0.719	0.723	0.774	0.733	0.834	0.760
Average Cervical Spine	0.897*	0.843	0.841	0.840	0.840	0.804
Total Average	0.761	0.726	0.772	0.779*	0.735	0.728

*Highest scores are in bold

Figure 3 also shows the comparison of cervical spine vertebral C.G rotational y-displacement. Overall, the cervical spine vertebral C.G rotations were better in the model without an active muscle controller, although oscillations were observed in C1-C5 without any muscle controller. Buckling (rapid changes in rotational velocites) was observed in C4 to C7 with all active muscle controllers except the Combined-Controller.

### Quantitative rating evaluation

All models with active muscle controllers improved overall head kinematics agreement with volunteer responses compared to the model without active muscle control (passive model) as shown by the CORA score values (Table 6, the highest scores were bold). The best overall head kinematics was found with the MLF controller only (0.719 /Good Rating). Meanwhile, for overall cervical spine kinematics agreement, the active muscle controller reduced the CORA score. The best CORA score among models with active muscle controllers was the model with only APF (0.841 /Good Rating). In addition, the model with either MLF or APF controllers improved the total kinematic agreement compared to the passive model. It should be noted that the passive model yielded the best CORA score for the cervical spine kinematics.

## Discussion

An attempt to combine and optimize the APF and MLF controllers was conducted in the present study. Two simplified approaches were proposed to combine the APF and MLF controller: one that assumed both APF and MLF controllers control all neck muscles together and another where APF controls the superficial muscles while deep neck muscles were controlled by the MLF controller. A high density of muscle spindles found in the deep cervical muscles motivated the second approach. In addition, optimization of the muscle co-contraction was proposed and applied to the model to keep the head upright under gravitational acceleration before implementing and optimizing the active muscle controllers.

Muscle co-contraction is defined as the simultaneous activation of agonistic and antagonistic muscles and known to contribute to maintaining spinal stability (Lee et al. 2006). Thus, under gravitational acceleration, muscle co-contraction should keep the neck stable and maintain a constant, upright, head position. In the present study, the neck muscles were initially divided into eight groups of muscles then the activation level (with a maximum of 5% from total activation level) of each muscle group was optimized with an objective to keep the head upright under gravitational loading. Neck muscle co-contractions should directly keep the head in an upright position and provide initial stiffness in the cervical spine. Previous implementations (Ólafsdóttir et al. 2019b; Putra et al. 2019, 2020) applied a global co-contraction level of 5% without ensuring this produced an equilibrium condition prior to applying external loads.

Even though muscle co-contraction levels were successfully optimized to keep the head upright under gravity loading, the assumption used in the current study was highly simplified. Posture maintenance under gravitational loading is complex and not yet fully understood as it involves different neural controls. Nevertheless, one study found that the neck muscle co-contractions could stiffen the joint and are necessary to provide stability at its neutral posture (Choi and Vanderby 2000). Optimized co-contractions could maintain the head in an upright position under gravity loading. A steady head position could be maintained after 200 ms and provides a good initial condition for simulating rear impacts.

Another reason to conduct optimization of the muscle co-contraction was that available experimental literature on the co-contraction ratios in neck muscles could not be directly applied to the model. The values available from the literature (such as from Choi 2003) did not directly reflect the activation level for Hill’s muscle model used in the current model. Therefore, a maximum of 5% was allowed in the optimizations to establish the minimum possible muscle co-contractions that could still meet the objective. The optimum co-contraction levels were then kept throughout the simulations (see Online Resource 2).

Combining APF and MLF controllers, either using combined-control or distributed approach, could not improve the head and neck kinematics agreement. These implementations produced the worst scores, even when compared to the passive model. When the activation signals of each controller were compared (Online Resource 2), the combined control always had the highest magnitude and was active earlier than the other strategies. This high magnitude could be because combining a vestibular inspired and muscle spindle feedback inspired controller was unsuitable using a simple minimum and maximum function. These investigated methods to incorporate the APF and MLF controllers can be considered pragmatic approaches. Several studies have implemented and combined VCR-like and CCR like controllers with various loading conditions (Ólafsdóttir et al. 2019b; Kleinbach 2019; Correia et al. 2021; Larsson et al. 2019). Ólafsdóttir et al. (2019b), Kleinbach (2019) and Larsson et al. (2019) assumed simple addition of the VCR-like and CCR-like controllers meanwhile Correia et al. (2021) assumed limited combination between VCR-like and CCR-like controller using IF function. The present study did not include a simple APF and MLF controller summation because the muscle activation signal could exceed 100% muscle activation. Simple addition could thus request muscle forces exceeding biomechanical limits and consequently lead to numerical instabilities.

In the closed-loop controller of Correia et al. (2021), the CCR-like controller was only implemented for the Trapezius and SCM muscle groups. In the present work, the CCR-like controller was implemented into all neck muscles (combined-control approach) or only deep muscles (distributed-control approach). In previous work (Correia et al. 2021), the maximum contribution of a CCR-like controller was only 10% of the total muscle force (based on Correia et al. (2020) defined using optimization). On the other hand, the CCR-like controller could activate 100% of the muscle forces in the present study. Based on the authors’ knowledge, there is no physiological reason why CCR reflexes could not fully activate neck muscles. Furthermore, Correia et al. (2021) used the IF function to activate the VCR-like controller if the head rotates more than five degrees, based on decerebrated cats' experiments. However, it is unclear that range the VCR reflex is active for the human head rotates. Therefore, in the current study, the VCR-like controller was kept active during the whole duration of simulation based on the well-known function of the VCR reflex itself, which is to maintain and stabilize head motion in space.

In the distributed approach, it was observed that the MLF controller had a minor influence on controlling the cervical vertebral rotation based on the change in deep muscle length. Therefore, it could not also improve the overall kinematics agreement with the volunteers.

Another important difference between the present study and previous studies (Ólafsdóttir et al. 2019b; Kleinbach 2019; Correia et al. 2021; Larsson et al. 2019) was that previous studies did not conduct any evaluations of intervertebral rotations of the cervical spine or use the intervertebral rotations as objectives in the optimization process to derive controller gains. The correct prediction of intervertebral rotation in the cervical spine is vital if a model will be used to study head-neck kinematics in whiplash-type motions.

Knowledge of the human sensory-motor system is still not well established (Keshner 2003; Armstrong et al. 2008; Goldberg and Cullen 2011; Cullen 2012). The human head-neck complex is controlled by separate systems that do communicate with each other. For example, Blouin et al. (2007) found that multifidus muscles (one of the deep neck muscles) have a focused spatial tuning curve, which is more similar to the APF controller than the MLF controller. The current implementations do not account for tactile or visual system feedback to the muscles. Therefore, further studies of the interdependency of APF and MLF characteristics are needed before separate muscle control systems can be successfully implemented in the same model. A challenge for this approach is the availability of information that allows specific reflex actions to be identified and quantified individually.

Another hypothesis why no significant improvements were observed in the kinematics agreement for the combined model implementations could be due to the limitations of the current Hill muscle implementation in LS-DYNA itself. As mentioned, and summarized in Kleinbach et al. (2017), the current implementation of LS Dyna Hill muscle only included a parallel damping element and neglected serial elastic and serial damping element to represent tendon structures.

The muscle in the current model was modeled as 1D Truss elements. Thus, it may lack damping effects from the 3D muscles containment. The damping value of 0.004 kN.ms/mm^2^ was initially used to define parallel damping elements and was based on Östh et al. (2012b). It was derived from simulations conducted to achieve reasonable damping compared to Hayes and Hatze (1977) experimental studies. However, this value may not be suitable for the present model as the study conducted by Östh et al. (2012b) was based on simulations of a human arm. The need for additional damping for the APF controller is highlighted by the much higher KD terms when compared to the MLF-based control. MLF seems to better control buckling behavior in the neck but is still not sufficient to achieve acceptable human-like responses.

Based on the overall simulation results, it was found that a single APF or MLF controller improved the head C.G displacements and cervical vertebral C.G rotation agreement with volunteers’ kinematics. Thus, it seems that either APF or MLF controller could be implemented to control neck muscle in a low-speed rear-impact scenario as it better controls the head C.G. displacement and cervical vertebral C.G. time histories than the passive model. However, due to the computational cost of the MLF controller and its complexity, as well as the fact each muscle element has its own PID controller, it seems strategic to use the APF controller for this purpose given the marginal difference in the CORA scores. This implementation will require further development if the head and neck kinematics are both to be well represented in a HBM that includes muscle forces.

The APF controller in the current study currently could not be used to conduct whiplash injury analysis. Global injury criteria such as Neck Injury Criteria (NIC) (Boström et al. 1996) or tissue-based injury assessment such as analysis of pressure gradients in the spinal canal (Svensson et al. 1998; Yao et al. 2016) and facet capsular ligament strains (Siegmund et al. 2001) should not be conducted using the current model with APF controller. This is due to the low agreement of head displacements between model and volunteer as well as oscillations that occurred in the lower cervical spine. This caused non-biofidelic responses and that would overpredict the pressure gradients in the spinal canal or strains of the cervical spine capsular ligament due to the non-representative cervical vertebral kinematics. These non-representative motions were observed despite that the average CORA score of cervical vertebral kinematics resulted in a good rating score. The challenge of optimizing both head and cervical spine kinematics simultaneously was also described by Putra et al. (2020) which found that the current model with APF controller could be tuned for each individual application (i.e., tuning global head kinematics to estimate NIC), but it will most probably produce non-biofidelic neck intervertebral responses. Therefore, further research to improve both the head and cervical vertebral kinematics of the model is needed before the model can be used for injury prediction analysis.

The present study also highlights the risk of studying neck injuries in an HBM that has been validated only for head kinematics. The results show that reasonable head kinematics can be reproduced even when the individual vertebrae are not reflecting biofidelic responses. This is also supported by previous work (Putra et al. 2020), which found that the calibrated and validated model for head kinematics only without included neck kinematics produced significant improvement in the head kinematics agreement with the volunteers; however, it sacrificed the agreement of neck kinematics.

In summary, the present study has contributed to a better understanding of how to model and calibrate neck muscle co-contractions, as well as the pros and cons of combining two muscle controllers in a single head-neck FE model. The intention was to create a robust model, not an exact duplicate of the human reflex system that cannot be fully validated. With future development, the present model could be used to study potential whiplash injuries mechanisms based on global kinematics or local tissue responses.

## Supplementary Information

Below is the link to the electronic supplementary material.

Supplementary file1 (DOCX 1385 kb)
