# Supplementary material for: Analysis of control strategies for VIVA OpenHBM with active reflexive neck muscles
Source: Biomech Model Mechanobiol. 2022 Aug 4;21(6):1731–42. doi: 10.1007/s10237-022-01616-y (PMC9700582; doi:10.1007/s10237-022-01616-y)

| **Name** | **Optimizations Objectives Function** | **Weight** | **Duration of Volunteer Data** | **Optimization Duration** |
| --- | --- | --- | --- | --- |
| CC+Co  (Combined-Control +  Co-Contraction) | To match volunteer head C.G horizontal x-disp. | 0.1 | 0-300ms | 200ms |
|  | To match volunteer head C.G rotational y-disp. | 0.1 | 0-300ms | 200ms |
|  | To match volunteer head C.G vertical y-disp. | 0.1 | 0-300ms | 200ms |
|  | To match volunteer C1 absolute rotational y-disp. | 0.1 | 0-180ms | 200ms (extrapolation) |
|  | To match volunteer C2 absolute rotational y-disp. | 0.1 | 0-180ms | 200ms (extrapolation) |
|  | To match volunteer C3 absolute rotational y-disp. | 0.1 | 0-180ms | 200ms (extrapolation) |
|  | To match volunteer C4 absolute rotational y-disp. | 0.1 | 0-180ms | 200ms (extrapolation) |
|  | To match volunteer C5 absolute rotational y-disp. | 0.1 | 0-180ms | 200ms (extrapolation) |
|  | To match volunteer C6 absolute rotational y-disp. | 0.1 | 0-180ms | 200ms (extrapolation) |
|  | To match volunteer C7 absolute rotational y-disp. | 0.1 | 0-180ms | 200ms (extrapolation) |
|  |  |  |  |  |
| DC+Co  (Distributed-Control +  Co-Contraction) | To match volunteer head C.G horizontal x-disp. | 0.1 | 0-300ms | 200ms |
|  | To match volunteer head C.G rotational y-disp. | 0.1 | 0-300ms | 200ms |
|  | To match volunteer head C.G vertical y-disp. | 0.1 | 0-300ms | 200ms |
|  | To match volunteer C1 absolute rotational y-disp. | 0.1 | 0-180ms | 200ms (extrapolation) |
|  | To match volunteer C2 absolute rotational y-disp. | 0.1 | 0-180ms | 200ms (extrapolation) |
|  | To match volunteer C3 absolute rotational y-disp. | 0.1 | 0-180ms | 200ms (extrapolation) |
|  | To match volunteer C4 absolute rotational y-disp. | 0.1 | 0-180ms | 200ms (extrapolation) |
|  | To match volunteer C5 absolute rotational y-disp. | 0.1 | 0-180ms | 200ms (extrapolation) |
|  | To match volunteer C6 absolute rotational y-disp. | 0.1 | 0-180ms | 200ms (extrapolation) |
|  | To match volunteer C7 absolute rotational y-disp. | 0.1 | 0-180ms | 200ms (extrapolation) |
|  |  |  |  |  |
| APF+Co  (Angular-positioned Feedback +  Co-Contraction) | To match volunteer head C.G horizontal x-disp. | 0.1 | 0-300ms | 200ms |
|  | To match volunteer head C.G rotational y-disp. | 0.1 | 0-300ms | 200ms |
|  | To match volunteer head C.G vertical y-disp. | 0.1 | 0-300ms | 200ms |
|  | To match volunteer C1 absolute rotational y-disp. | 0.1 | 0-180ms | 200ms (extrapolation) |
|  | To match volunteer C2 absolute rotational y-disp. | 0.1 | 0-180ms | 200ms (extrapolation) |
|  | To match volunteer C3 absolute rotational y-disp. | 0.1 | 0-180ms | 200ms (extrapolation) |
|  | To match volunteer C4 absolute rotational y-disp. | 0.1 | 0-180ms | 200ms (extrapolation) |
|  | To match volunteer C5 absolute rotational y-disp. | 0.1 | 0-180ms | 200ms (extrapolation) |
|  | To match volunteer C6 absolute rotational y-disp. | 0.1 | 0-180ms | 200ms (extrapolation) |
|  | To match volunteer C7 absolute rotational y-disp. | 0.1 | 0-180ms | 200ms (extrapolation) |
|  |  |  |  |  |
| MLF+Co  (Muscle-length Feedback +  Co-Contraction) | To match volunteer head C.G horizontal x-disp. | 0.1 | 0-300ms | 200ms |
|  | To match volunteer head C.G rotational y-disp. | 0.1 | 0-300ms | 200ms |
|  | To match volunteer head C.G vertical y-disp. | 0.1 | 0-300ms | 200ms |
|  | To match volunteer C1 absolute rotational y-disp. | 0.1 | 0-180ms | 200ms (extrapolation) |
|  | To match volunteer C2 absolute rotational y-disp. | 0.1 | 0-180ms | 200ms (extrapolation) |
|  | To match volunteer C3 absolute rotational y-disp. | 0.1 | 0-180ms | 200ms (extrapolation) |
|  | To match volunteer C4 absolute rotational y-disp. | 0.1 | 0-180ms | 200ms (extrapolation) |
|  | To match volunteer C5 absolute rotational y-disp. | 0.1 | 0-180ms | 200ms (extrapolation) |
|  | To match volunteer C6 absolute rotational y-disp. | 0.1 | 0-180ms | 200ms (extrapolation) |
|  | To match volunteer C7 absolute rotational y-disp. | 0.1 | 0-180ms | 200ms (extrapolation) |

**Online Resource 1. Active Muscle Controller Optimization**

| **Online Resource 2. Comparison of Muscle Activation Signals between Controllers.** | |
| --- | --- |
| 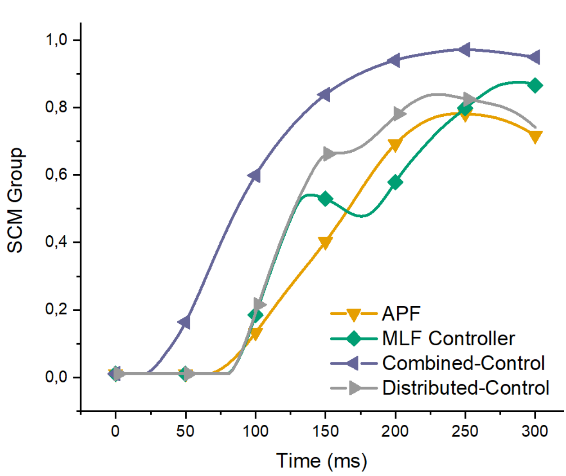 | 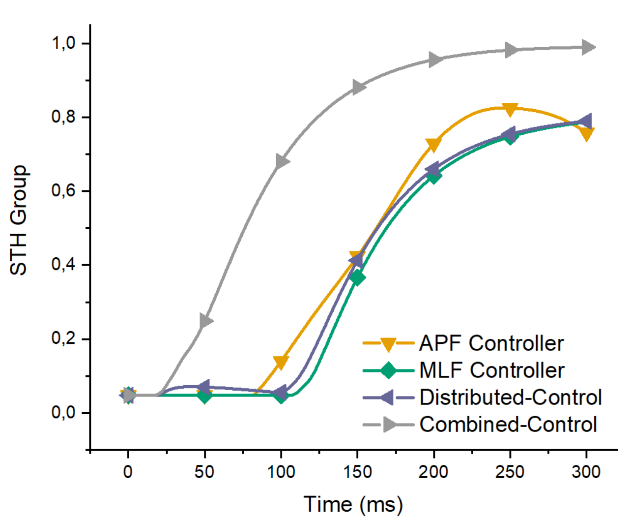 |
| 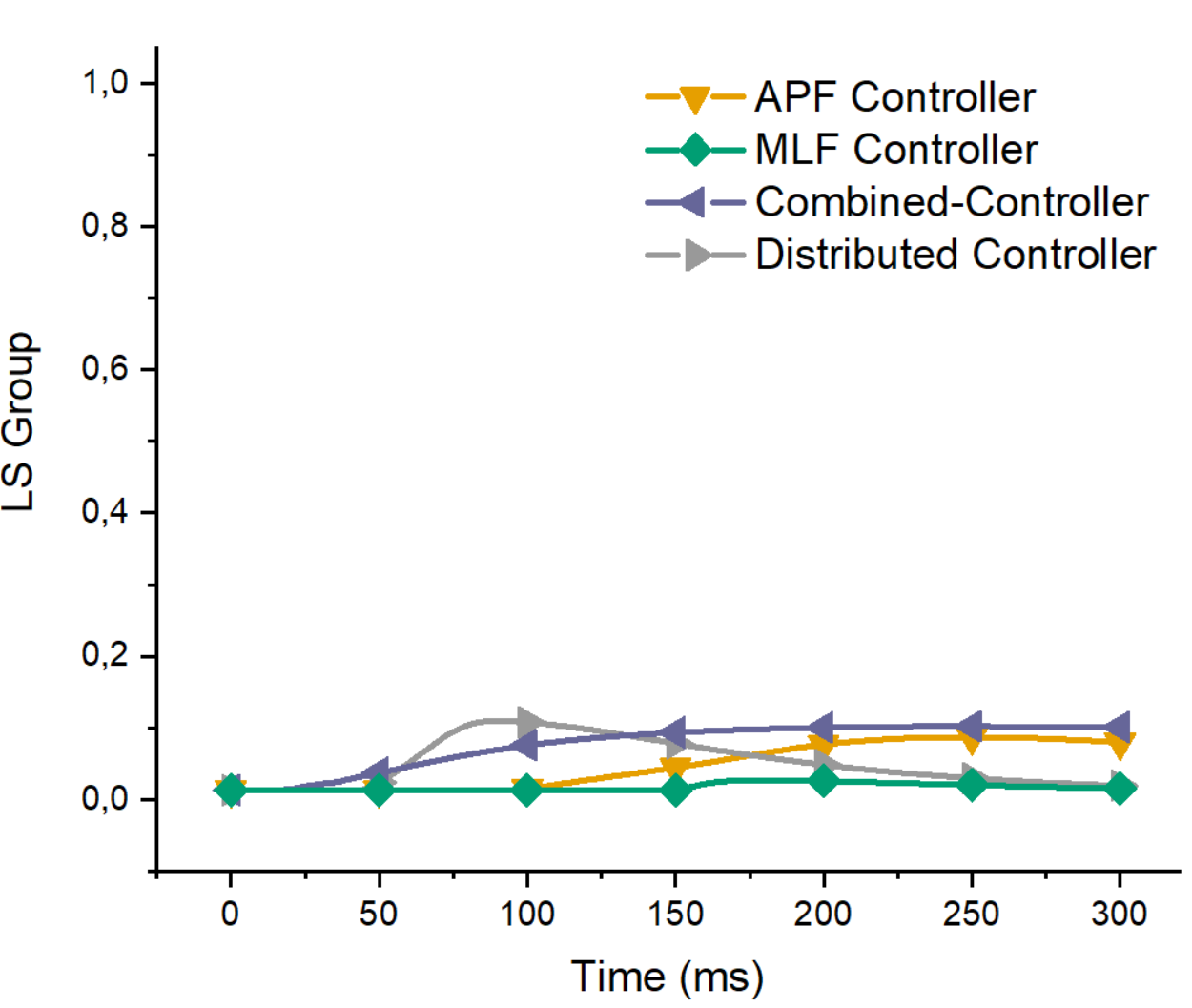 | 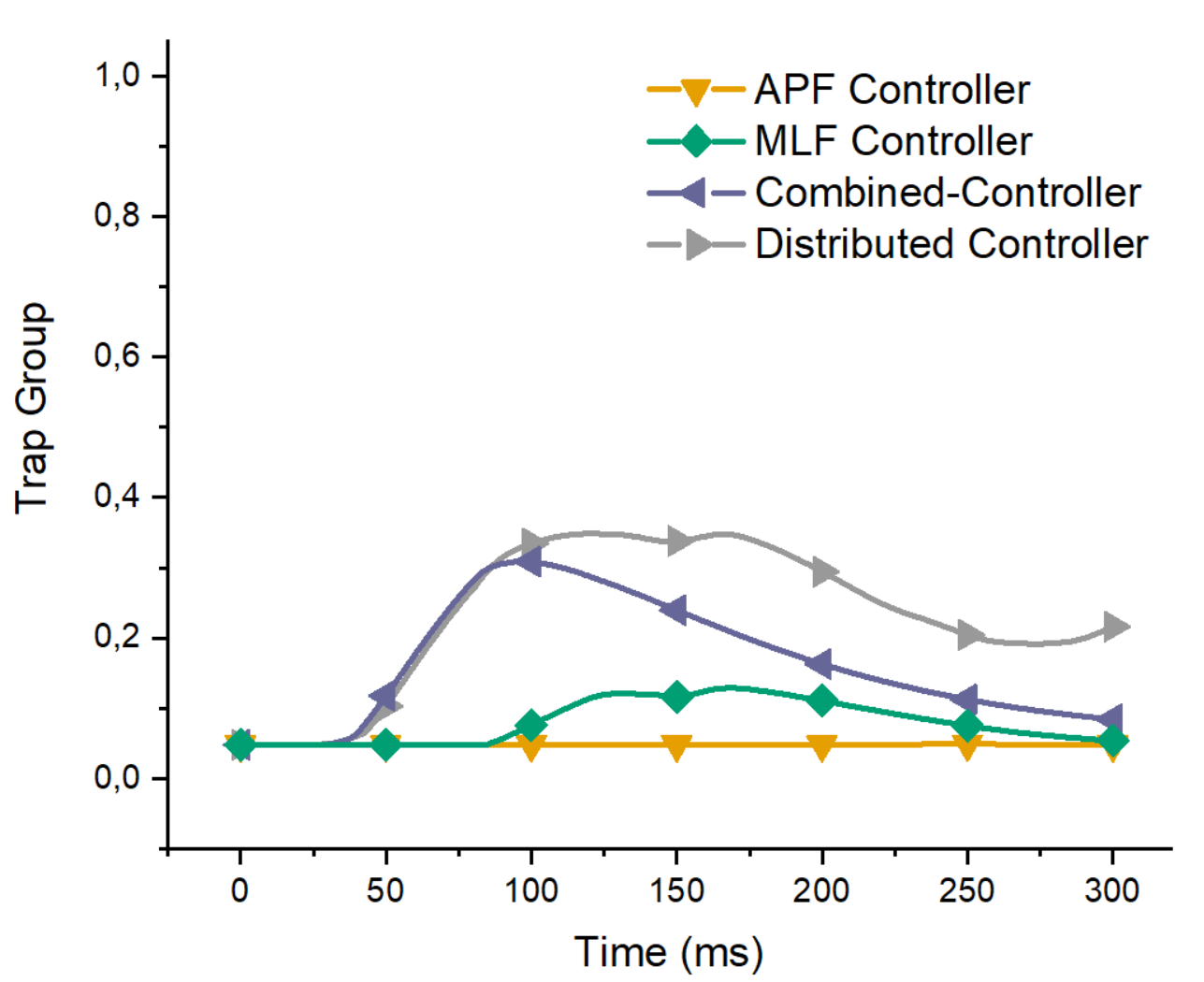 |
| 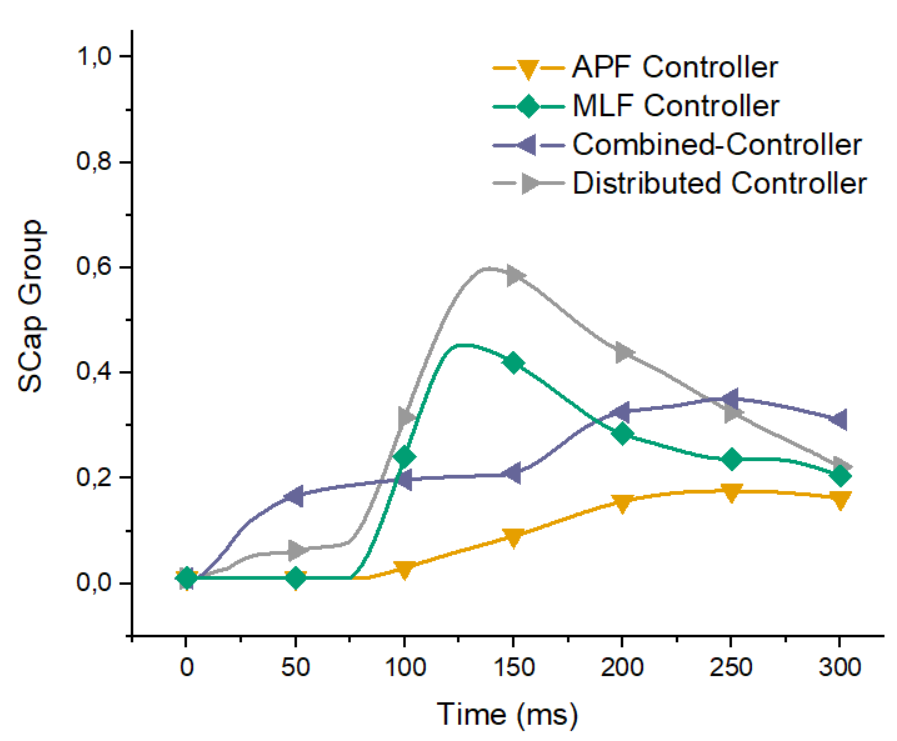 | 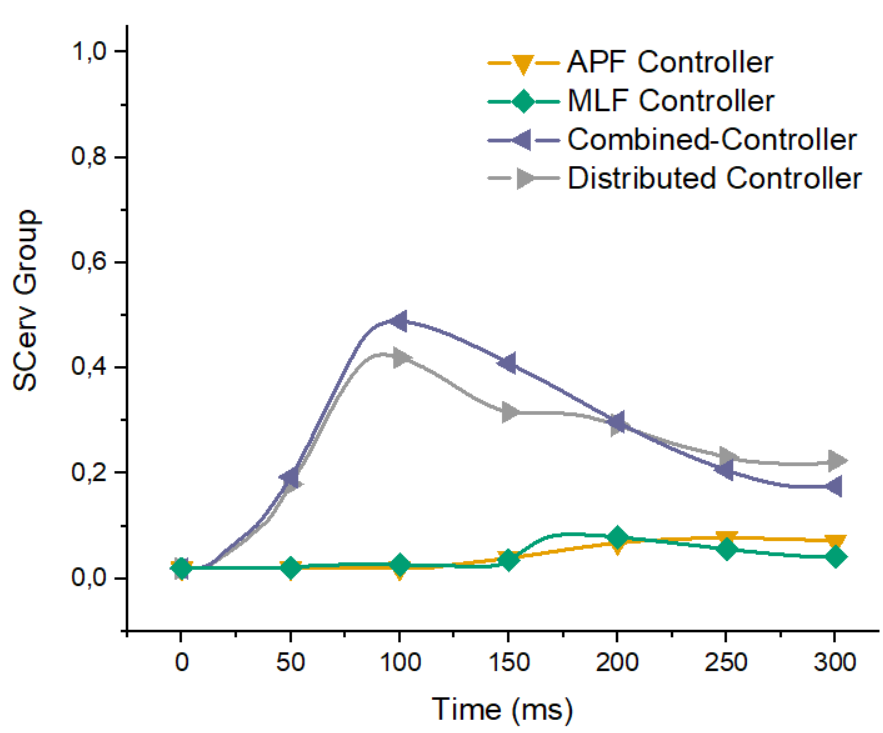 |
| 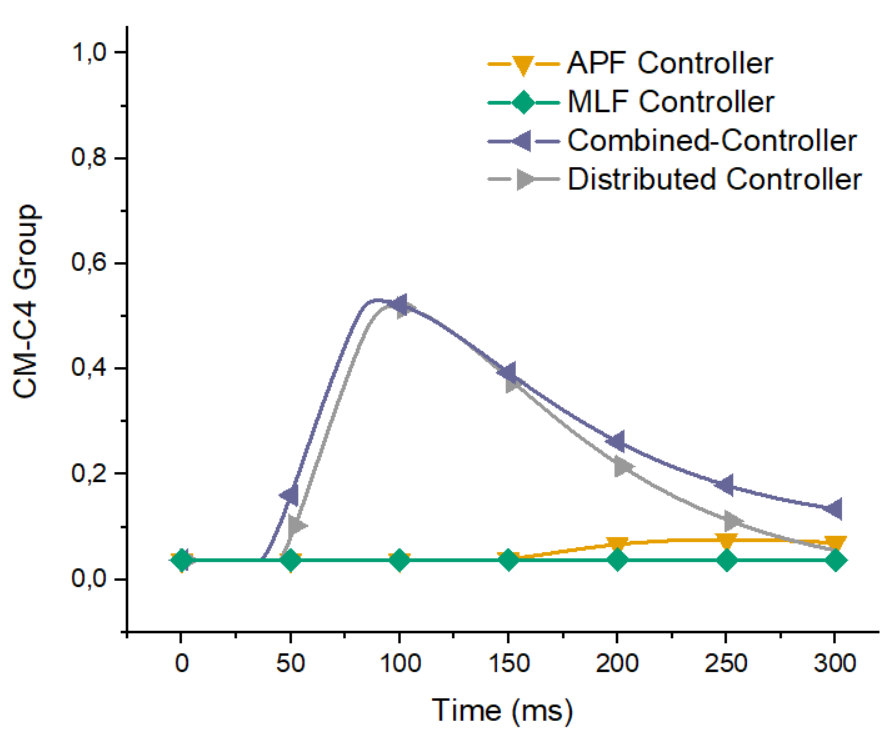 | 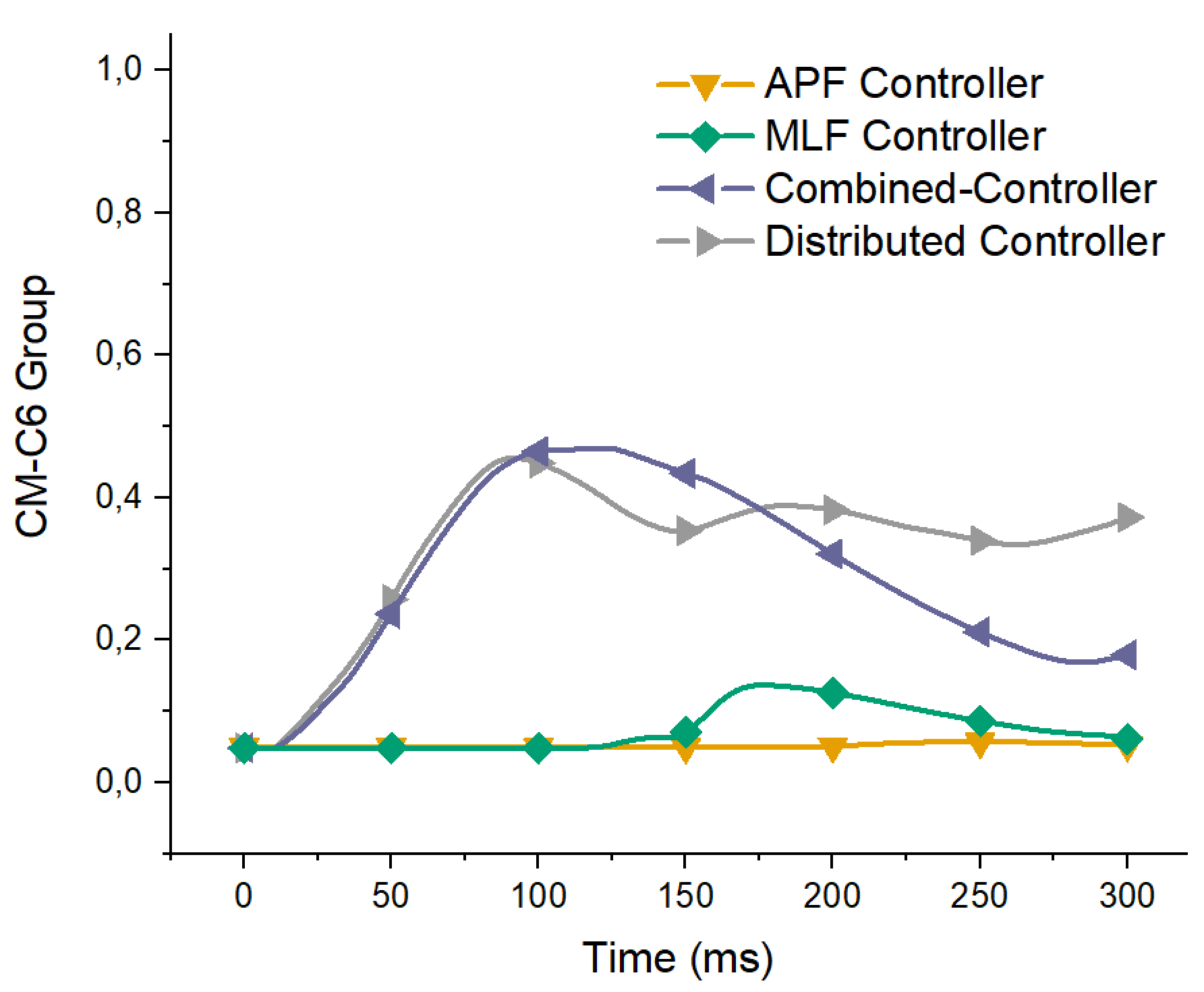 |

| **Online Resource 3. Comparison of Head C.G Accelerations between VIVA OpenHBM and Referenced Volunteer (Sato et al. 2014)** |
| --- |


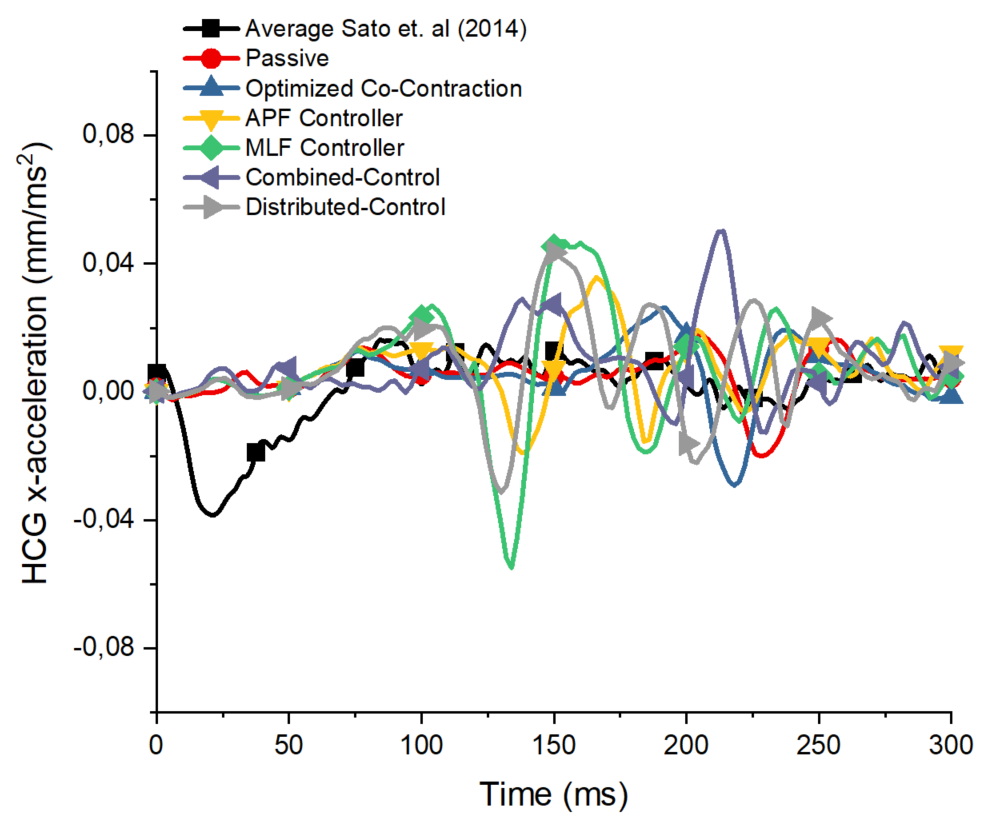

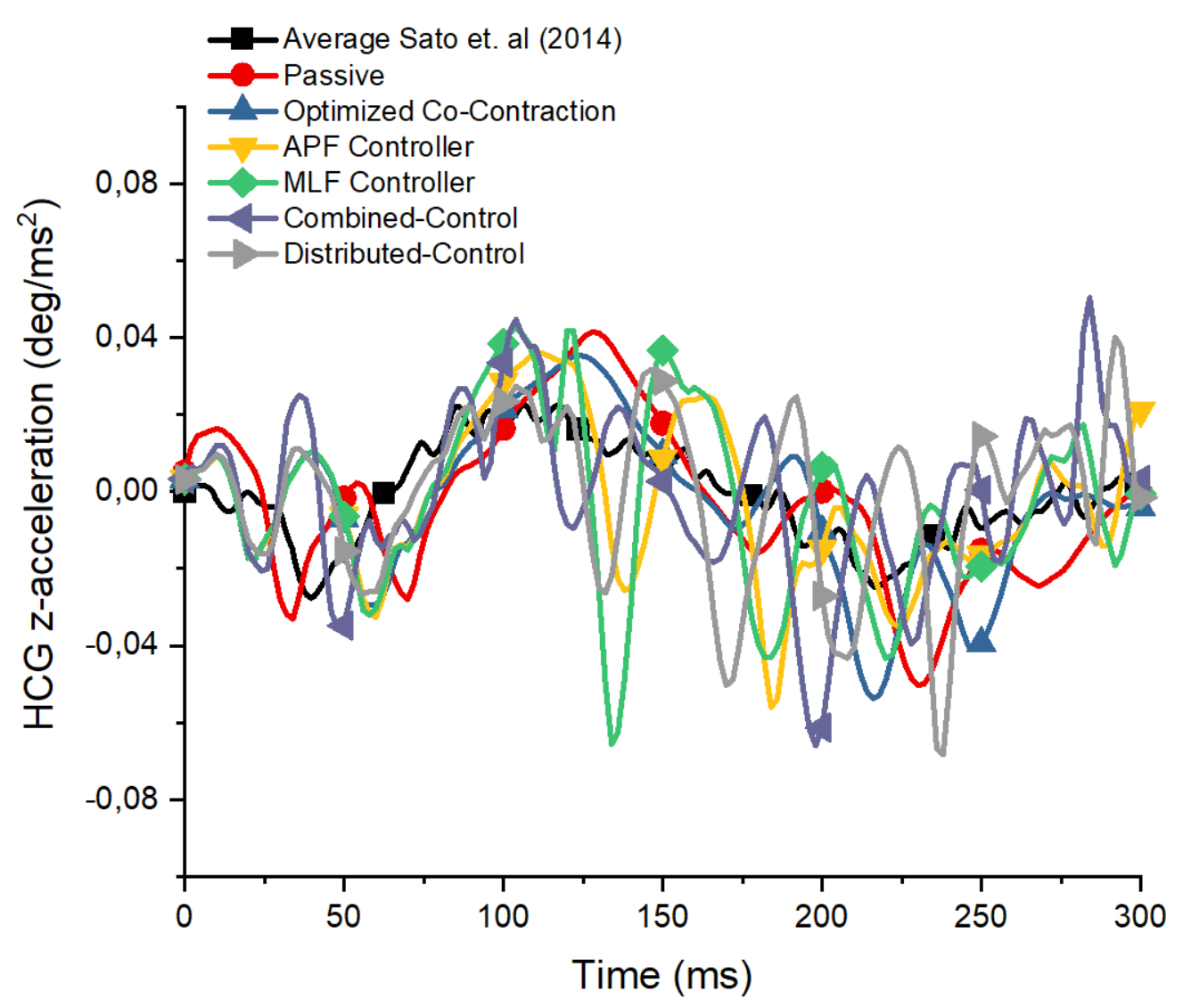

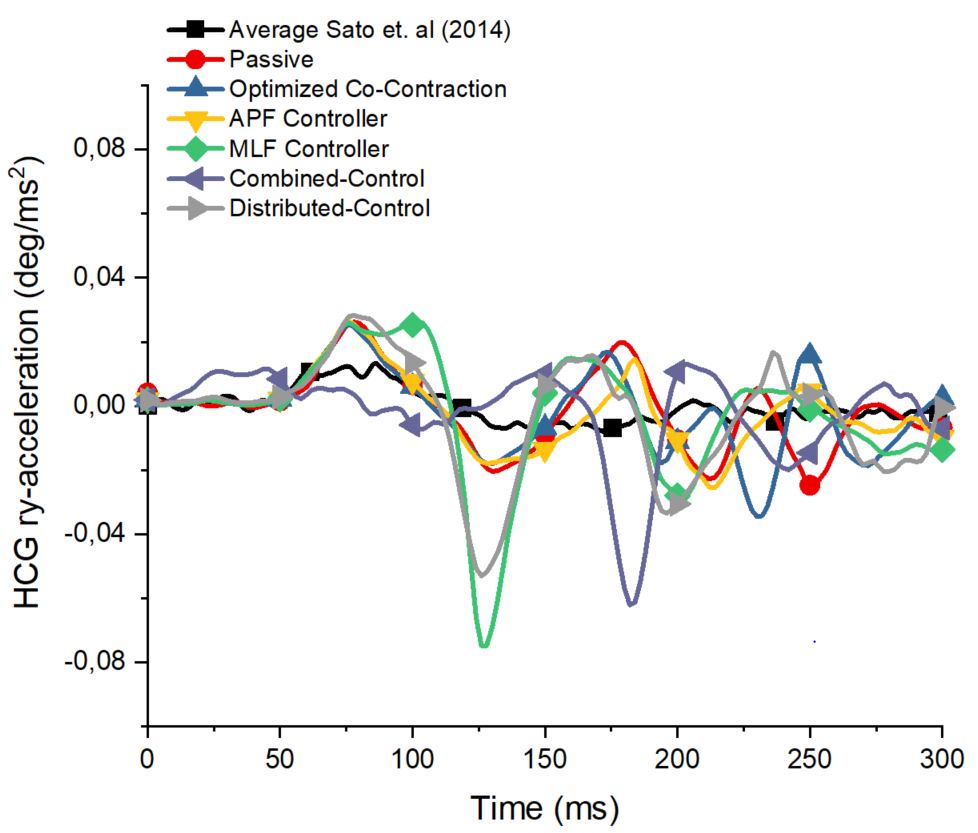

Supplement: Supplementary file 1 — Supplementary file1 (DOCX 1385 kb) [file 10237_2022_1616_MOESM1_ESM.docx]
